# Effect of Empagliflozin and Pioglitazone on left ventricular function in patients with type two diabetes and nonalcoholic fatty liver disease without established cardiovascular disease: a randomized single-blind clinical trial

**DOI:** 10.1186/s12876-023-02948-4

**Published:** 2023-09-23

**Authors:** Fereshte Attaran, Sepideh Emami, Masoudreza Sohrabi, Mojtaba Malek, Hossein Ajdarkosh, Mahmoodreza Khoonsari, Faramarz Ismail-Beigi, Mohammad E. Khamseh

**Affiliations:** 1 Endocrine Research Center, Institute of Endocrinology and Metabolism, Department of Internal Medicine, School of Medicine, Iran University of Medical Science, No. 10, Firoozeh St., Vali-asr Ave., Vali-asr Sq, Tehran, Iran; 2 Department of Cardiology, Firoozgar Hospital, School of Medicine, Iran University of Medical Sciences, Tehran, Iran; 3 Gastrointestinal and liver diseases research center, Iran University of Medical Sciences, Tehran, Iran; 4 Research Center for Prevention of Cardiovascular Disease, Institute of Endocrinology and Metabolism, Iran University of Medical Sciences, Tehran, Iran; 5 School of Medicine, Case Western Reserve University, Cleveland, USA; 6 Endocrine Research Center, Institute of Endocrinology and Metabolism, Iran University of Medical Science, Tehran, Iran

**Keywords:** Empagliflozin, Pioglitazone, Ventricular function, Liver cirrhosis, Non-alcoholic fatty liver disease, Heart, heart failure, Clinical trial

## Abstract

**Background:**

Nonalcoholic fatty liver disease (NAFLD) is a complex metabolic disorder that increases the risk for cardiovascular disease in patients with type 2 diabetes mellitus (T2DM). Global longitudinal strain (GLS) is an indicator of left ventricular (LV) mechanics and can detect subclinical myocardial dysfunction. We compared the effects of pioglitazone and empagliflozin on GLS in patients with T2DM and NAFLD without established atherosclerotic cardiovascular disease.

**Methods:**

This study was a 24-week randomized, single-blind, and parallel-group (1: 1 ratio) clinical trial. Seventy-three participants with T2DM (being treated with metformin) and NAFLD but without established atherosclerotic cardiovascular disease (ASCVD) were randomized to empagliflozin or pioglitazone. Liver steatosis and fibrosis were measured using transient elastography, and GLS was measured by echocardiography. The primary endpoint was the change in GLS from baseline to week 24. Secondary end points include changes in controlled attenuation parameter (CAP) and Liver stiffness measure (LSM).

**Results:**

In this study, GLS improved by 1.56 ± 2.34% (P < 0.01) in the pioglitazone group and 1.06 ± 1.83% (P < 0.01) in the empagliflozin group without a significant difference between the two groups (P = 0.31). At baseline, GLS was inversely associated with the severity of liver fibrosis: r = − 0.311, P = 0.007. LSM in the pioglitazone and empagliflozin group [(-0.73 ± 1.59) and (-1.11 ± 1.33)] kpa (P < 0.01) decreased significantly. It was without substantial difference between the two groups (P = 0.26). Empagliflozin and pioglitazone both improved controlled attenuation parameter. The improvement was more critical in the empagliflozin group: -48.22 + 35.02 dB/m vs. -25.67 + 41.50 dB/m, P = 0.01.

**Conclusion:**

Subclinical cardiac dysfunction is highly important in patients with T2DM and with NAFLD. Empagliflozin and Pioglitazone improve LV mechanics and fibrosis in patients without established ASCVD. This has a prognostic importance on cardiovascular outcomes in high-risk patients with T2DM. Moreover, empagliflozin ameliorates liver steatosis more effectively them pioglitazone. This study can serve as a start point hypothesis for the future. Further studies are needed to explore the concept in larger populations.

**Trial registration:**

: This trial was registered in the Iranian Registry of Clinical Trials (IRCT): “A Comparison between the Effect of Empagliflozin and Pioglitazone on Echocardiographic Indices in Patients with Type 2 Diabetes Mellitus and Nonalcoholic Fatty Liver Disease” IRCT20190122042450N5, 29 November 2020. https://www.irct.ir/search/result?query=IRCT20190122042450N5.

**Supplementary Information:**

The online version contains supplementary material available at 10.1186/s12876-023-02948-4.

## Background

The prevalence of Nonalcoholic fatty liver disease (NAFLD) is increasing in parallel with the worldwide increase in obesity and type 2 diabetes mellitus (T2DM) [1]. NAFLD is associated with extra-hepatic metabolic comorbidities, high blood pressure, hyperlipidemia, and cardiovascular disease (CVD) [2–4]. There are common pathophysiological mechanisms shared between NAFLD and T2DM [5]. Hence, NAFLD could potentially increase the risk of heart failure and mortality in patients with T2DM. Earlier studies have shown that anti-hyperglycemic agents can improve abnormal liver enzymes [6–8]. In addition, pioglitazone and empagliflozin can decrease hepatic steatosis [9, 10].

The relationship between NAFLD and CVD is complex [11], and NAFLD is considered an independent risk factor for CVD and heart failure [12]. Recent studies demonstrated that NAFLD is associated with structural heart disease and myocardial functional abnormalities [13, 14]. These abnormalities can be evaluated by imaging techniques that measure myocardial strain, including complete two-dimensional color doppler echocardiography and magnetic resonance imaging (MRI) [15].

Global longitudinal strain (GLS) is one of the most sensitive and accurate echocardiographic measures for assessing LV mechanics and determining subclinical left ventricular systolic dysfunction [16]. In addition to systolic dysfunction, NAFLD is also associated with diastolic cardiac dysfunction [13, 17].

To the best of our knowledge, no previous study has compared the effects of empagliflozin and pioglitazone on subclinical cardiac dysfunction that is associated with NAFLD. The present study was designed to compare the impact of these two agents on echocardiographic indices in individuals with T2DM and NAFLD who did not have established atherosclerotic cardiovascular disease (ASCVD).

## Methods

### Study design

This study was a 24-week prospective, 1:1 ratio randomized, single-blind clinical trial. It was registered in the Iranian Registry of Clinical Trials (IRCT): IRCT20190122042450N3. The study protocol was approved by the Ethics Committee (EC) of the Institutional Review Board (IRB) of the Iran University of Medical Science; ethical code: IR.IUMS.REC.1398.1408. Iran University of Medical Science (IUMS) funded the study. All of the participants signed a written informed consent before enrollment and after a face-to-face discussion about the study procedures and outcomes. Abidi pharmaceutical company supplied the medication. However, they had no role in data management, analyses, and writing final report.

### Participants

We invited patients with T2DM aged 20 to 80 to participate in this study. In a 3-week run-in period, volunteers were screened using standard medical history, biochemical tests, liver elastography, and Echocardiography (Supplement 1).

Patients with HbA1c greater than 7% and less than 10.5%, a controlled attenuation parameter (CAP) of ≥ 302 dB/m [18], and without established ASCVD were recruited. All the participants were on standard antidiabetic therapy at least six months prior to enrollment. Established ASCVD was defined as having documented previous history of at least one of the following at screening: acute myocardial infarction, ischemic stroke, peripheral artery disease, coronary revascularization, and hospitalization for heart failure. The exclusion criteria were: EF < 50%, current use of sodium- glucose cotransporter-2 (SGLT2) inhibitors, glucagon-like peptide 1 receptor agonists, thiazolidinediones, tamoxifen, amiodarone, non-steroidal anti-inflammatory drugs (NSAIDs), vitamin C, vitamin E, selenium, and antioxidants, pregnancy or breastfeeding, uncontrolled hypothyroidism or hyperthyroidism, acute viral hepatitis, autoimmune hepatitis, estimated glomerular filtration rate (eGFR) < 45 mL/min/1.73 m^2^, active cancer, clinical signs of cirrhosis, and alcohol consumption more than 20 g/day in females or greater than 30 g/day in males for at least three consecutive months over the past five years. All patients were asked to follow standard lifestyle modification recommendations according to the American Diabetes Association’s (ADA’s) Standards of Medical Care in Diabetes [19].

### Randomization

A computerized block randomization method was used for randomization. Participants were randomized to receive pioglitazone 30 mg/day or empagliflozin 10 mg/day for 24 weeks. The intervention medications were sealed sequentially and numbered according to the allocation sequence. An independent staff generated the random allocation sequence and assigned participants to the study arms. The care provider was blinded to the intervention. Medications were supplied free of charge.

### Procedures

Blood samples were collected after 8 to 12 h of fasting. Lipid profile, fasting blood glucose, liver enzymes, and serum creatinine were measured by Pars biochemical kits using the photometric method. HbA1c was measured using capillary method. Markers for viral hepatitis were also assessed.

Antinuclear antibodies (ANA) was using immunofluorescence method. ELISA was used to measure thyroid hormones. Triglyceride-glucose index (TyG index), a measure of insulin resistance (IR), was calculated using the formula: (TyG) = Ln [FBS (mg/dl) × TG (mg/dl)/2] [20].

Liver elastography was performed at baseline and the end of the study. Liver fibrosis was defined according to the liver stiffness measurement (LSM).

Hepatic steatosis was measured based on the controlled attenuation parameter (CAP) using Fibroscan® 502 Touch equipped with both M and XL probes. The procedure was performed by an expert physician (blinded to the study protocol) trained and certified by the Iranian Association of Gastroenterology and Hepatology (IAGH). Participants with CAP greater than 302 dB/m were enrolled in the study.

### Echocardiography evaluation

Transthoracic Echocardiography (TTE) was performed using echocardiography equipment, Acuson SC2000TM ultrasound system (Acuson, Siemens, CA, USA), with a 4 MHz probe (Acuson 4V1c). The images were obtained while subjects were at rest and in the left lateral decubitus position. All images acquisition and measurement were performed according to the recommendations of the American Society of Echocardiography (ASE) guidelines.

Standard 2D-TTE and strain imaging were performed at baseline and week 24 by an expert cardiologist who was blinded to the study protocol. Two-dimensional images were optimized to achieve the highest frame rate possible. Tissue Doppler Imaging (TDI) of the interventricular septum and lateral wall was recorded using the apical 4-chamber view with the narrowest sector angle feasible and a high frame rate. The images were digitally captured for subsequent analysis. LV systolic and diastolic function parameters including the global LV ejection fraction (LV EF) were assessed. LV EF was calculated by modified Simpson’s rule after measuring the end-diastolic volume (EDV) and the end-systolic volume (ESV), from both four-chamber and two-chamber views. LV diastolic function was assessed via pulsed-wave (PW) Doppler of the mitral inflow. Indices were measured by placing the pulsed-wave Doppler at the tip of the mitral valve leaflets during diastole, and mitral inflow velocities were recorded from the apical 4-chamber view (A4C). These included measurements of peak E-wave velocity (E), peak A-wave velocity (A), mitral E/A ratio, mitral E-wave deceleration time (DT), and isovolumic relaxation time (IVRT), which was recorded as the interval between aortic valve closure and mitral valve opening at a sweep speed of 100 mm/s. The Tissue Doppler Imaging (TDI)-driven mitral annular e’ velocity was measured as the average of septal and lateral e’ velocities. The E/e’ ratio was calculated as an index of LV filling pressure. We also measured LA volume at end of systole using Simpson rule. It was indexed for body surface area to drive LAVi. In addition, estimated pulmonary systolic pressure was determined.

### Speckle tracking and global longitudinal strain(GLS) analysis

Strain imaging was performed semi-automatically. Three standard apical views were recorded (apical four-chamber, apical two-chamber, and apical three-chamber) at the end of expiration in three consecutive cardiac cycles, with a frame rate ranging between 40 and 60 frames per second. Offline analysis was conducted using approved software available on the Acuson ultrasound equipment. Endocardial borders were meticulously traced manually in each image. Aortic valve closure was automatically identified by the software and edited if necessary. Longitudinal strain values were calculated by the software and presented in the bull’s-eye tomogram, numeric values and as the average global longitudinal strain (GLS) among the 16 segments. GLS was measured in two cardiac cycles, and the average value was used for the final analysis. The normal value for GLS was considered as the absolute value greater than 18% [21].

### Follow up

Follow-up visits include in-person visits at baseline and weeks 12 and 24. Remote visits via phone were done by week 4, 8, 16, and 20. Anti-hyperglycemic treatments were changed, as necessary, according to the recommendations of the American Diabetes Association Guideline (ADA) 2019. Before enrollment, moderate-intensity statin was initiated for patients who were not receiving a statin, according to the American Diabetes Association’s (ADA’s) Standards of Medical Care in Diabetes [19]. The dose of empagliflozin and pioglitazone remained constant during the study.

The International Physical Activity Questionnaire (IPAQ) assessed the participants’ physical activity [22]. Patient adherence and adverse events were recorded during in-patient visits.

### Outcomes measured

The primary outcome was the change in GLS compared to the baseline. Secondary outcomes included changes in diastolic parameters as well as changes in CAP, LSM, fibrosis-4 (FIB4) index, and NAFLD fibrosis score from the baseline.

### Statistical analysis

Sample size was calculated based on the study of Akelbrom A et al., which explored the effect of dapagliflozin on GLS in patients with T2DM [23]. To indicate a difference of 1.5% in GLS with a power of 80%, a total number of 70 subjects were calculated to be enrolled in the study, considering a 20% dropout rate in each treatment group.

Baseline characteristics are shown as means ± standard deviation (SD) for continuous variables and percentages for categorical variables. Within-group changes from baseline were examined by paired t-test. We used analysis of variance (ANOVA) to assess the between-group comparisons. Correlations were evaluated by Pearson’s correlation test for parametric variables and Spearman’s correlation test for nonparametric variables. One participant in the pioglitazone group and two in the empagliflozin group withdrew during the treatment period. Analysis of the data was by intention to treat (ITT) method. P-values less than 0.05 were considered statistically significant. The analyses were done using SPSS software version 25.0.

## Results

Seventy three participants (37 patients in the empagliflozin group and 36 patients in the pioglitazone group) were enrolled to the two study arms. Data from these patients were used for an intention to treatment analysis. Three patients (one in the pioglitazone group and two in the empagliflozin group) did not complete the study due to non-adherence to the study medications. The flowchart of patient enrollment is shown in Supplement 2. The baseline characteristics of the participants are shown in Table 1. In the entire cohort at study entry, there was a significant correlation between GLS and LMS (r = − 0.311, P = 0.007), but there was no statistically significant correlation between GLS and CAP (r = − 0.227, P = 0.053). At baseline, there were no statistically significant differences between the two groups regarding age, sex, body mass index (BMI), diabetes duration, and HbA1c (Table 1). Concurrent drug treatment at baseline is shown in Supplement 3. There was no statistically significant difference between the two groups in relation to background treatment for diabetes, hypertension and hyperlipidemia.

**Table 1:** Baseline characteristics of the participants

	Pioglitazone Group (n = 36)	Empagliflozin Group (n = 37)	P value^**^
Age (yrs.)	52 ± 7	52 ± 7	0.74
Female (%)	58.3%	54.0%	0.71
BMI (kg/m^2^)	31.3 ± 5.0	31.0 ± 3.8	0.80
Diabetes duration (yrs.)	7.9 ± 5.3 (1.5–24)	8.0 ± 6.1 (0.5–30)	0.88
FBS (mg/dl)	165 ± 49	177.57 ± 66.40	0.37
HbA1c (%)	8.4 ± 1.1	8.6 ± 1.1	0.47
TG (mg/dl)	212.8 ± 127.9	161.7 ± 70.6	0.04
Tchol (mg/dl)	158.3 ± 32.4	146.0 ± 30.3	0.10
LDL-C (mg/dl)	100.1 ± 20.6	96.3 ± 26.0	0.49
HDL-C (mg/dl)	46.7 ± 9.3	44.8 ± 8.9	0.37
ALT (IU/L)	31.5 ± 12.2	31.8 ± 17.6	0.93
AST (IU/L)	23.0 ± 7.7	25.1 ± 14.5	0.43
TyG*	9.6 ± 0.6	9.4 ± 0.7	0.26
CAP (dB/m)	329.8 ± 19.1	325.1 ± 18.4	0.29
LSM (kPa)	7.2 ± 2.5	7.4 ± 3.4	0.75

### Effect of pioglitazone and empagliflozin on GLS

At baseline, GLS was − 19.07 ± 2.40% and − 19.65 ± 2.15% in the pioglitazone and empagliflozin group, respectively with no difference between the two (P = 0.28). There was an inverse linear relationship between the GLS and liver fibrosis (LSM) at baseline (r = − 0.311, P = 0.007). We found statistically significant improvement in GLS in both treatment groups. GLS improved by (1.57 + 2.34%) and (1.07 + 1.83%) in the pioglitazone and empagliflozin group respectively, (P < 0.01). It was no statistically significant difference between the two groups (P = 0.31). In addition, there was no linear correlation between the improvement of GLS and the changes in the LSM.

### Effect of pioglitazone and empagliflozin on liver steatosis and fibrosis

After 24 weeks, liver steatosis was improved by 25.6 ± 41.5 dB/m (P < 0.01) in the pioglitazone group and by 48.2 ± 35.0 dB/m (P < 0.01) in the empagliflozin group. Empagliflozin was more effective than pioglitazone in reducing hepatic steatosis (P = 0.01). Furthermore, both pioglitazone and empagliflozin improved liver fibrosis by 0.7 ± 1.5 kpa, and 1.1 ± 1.3 kpa, respectively (Table 2).

**Table 2:** Liver Steatosis and Fibrosis at baseline and by week 24

Variable	Pioglitazone Group (n = 36)			Empagliflozin Group (n = 37)			
	Baseline	After 24 weeks	P value	Baseline	After 24 weeks	P value	P value*
CAP (dB/m)	329.8 ± 19.1	304.1 ± 32.8	< 0.01	325.1 ± 18.4	276.9 ± 39.0	< 0.01	0.01
LSM (kPa)	7.2 ± 2.5	6.4 ± 1.5	0.01	7.4 ± 3.4	6.3 ± 2.4	< 0.01	0.26

CAP, controlled attenuation parameter; LSM, Liver stiffness measurement

*Between-group difference from baseline, P < 0.05 is significant.

### Effect of pioglitazone and empagliflozin on hemodynamics and echocardiographic indices

Systolic blood pressure was reduced by 3.57 ± 8.18 mmHg in the empagliflozin group (P-value = 0.01). However, the reduction was insignificant in the pioglitazone group (P-value = 0.23). Diastolic blood pressure was reduced in both groups with no statistically significant difference between the two groups. EF at baseline was 58.7% ± 8.1% and 57.9% ± 6.9% in the pioglitazone and empagliflozin respectively.

The reduction of the E/e’ ratio was not significant in the pioglitazone group: 9.76 ± 2.08 at baseline and 9.45 ± 1.68 by the end of the study (P = 0.39). On the other hand, it decreased significantly from 9.93 ± 2.49 to 9.09 ± 1.89 (P = 0.04) in the empagliflozin group. Pioglitazone and empagliflozin did not affect cardiac dimensions and pulmonary artery pressure after 24 weeks (Table 3).

**Table 3:** Hemodynamics and Echocardiographic Indices at Baseline and by week 24

	Pioglitazone Group (n = 36)			Empagliflozin Group (n = 37)			
**Variable**	**Baseline**	**After 24 weeks**	**P value**	**Baseline**	**After 24 weeks**	**P value**	P value*
Systolic (mmHg)	117.5 ± 14.5	114.8 ± 11.7	0.23	116.8 ± 11.8	113.5 ± 11.3	0.01	0.80
Diastolic (mmHg)	75.2 ± 11.3	71.2 ± 8.2	0.03	77.5 ± 9.4	72.5 ± 9.1	< 0.01	0.62
EF (%)	58.7 ± 8.1	58.7 ± 4.5	0.99	57.9 ± 6.9	59.1 ± 6.5	0.38	0.55
GLS (%)	-19.07 ± 2.40	-20.64 ± 1.72	< 0.01	-19.65 ± 2.15	-20.72 ± 1.56	< 0.01	0.31
E (cm/s)	0.66 ± 0.11	0.71 ± 0.13	0.01	0.64 ± 0.12	0.66 ± 0.11	0.50	0.14
A (cm/s)	0.68 ± 0.13	0.71 ± 0.13	0.04	0.67 ± 0.16	0.69 ± 0.16	0.38	0.48
E/A	1.00 ± 0.26	1.04 ± 0.29	0.31	1.00 + 0.29	0.99 + 0.27	0.87	0.51
e´ (cm/s)	6.8 ± 1.5	7.5 ± 1.6	0.01	6.5 ± 1.5	7.3 ± 1.7	< 0.01	0.82
E/ e´	9.7 ± 2.0	9.4 ± 1.6	0.39	9.9 ± 2.4	9.0 ± 1.8	0.04	0.31
DT (ms)	221.9 ± 39.5	205.9 ± 52.1	0.12	201.0 ± 33.4	213.0 ± 48.8	0.13	0.03
IVRT (ms)	108.1 ± 13.8	100.0 ± 15.7	0.02	107.1 ± 15.9	106.9 ± 13.7	0.95	0.07
LV end-diastolic diameter (cm)	4.4 ± 0.4	4.4 ± 0.4	0.79	4.6 ± 0.4	4.5 ± 0.4	0.20	0.42
LV end-systolic diameter (cm)	2.9 ± 0.4	2.8 ± 0.3	0.17	3.1 ± 0.4	2.9 ± 0.4	0.10	0.84
LAVi (ml/m^2^)	23.0 ± 4.1	24.2 ± 5.0	0.28	21.7 ± 6.2	21.1 ± 1.1	0.40	0.16
LA area (cm^2^)	15.7 ± 2.3	16.3 ± 2.1	0.10	15.4 ± 2.5	15.6 ± 2.2	0.59	0.43
PAP (mmHg)	24.9 ± 3.3	25.1 ± 3.1	0.86	24.9 ± 3.3	25.2 ± 3.3	0.72	0.92

A, A wave; DT, deceleration time; E, E wave; e’, e’ wave; EF, ejection fraction; E/A, E to A ratio; GLS, global longitudinal strain; IVRT, Isovolumic relaxation time; LA, left atrium; LAVi, left atrium volume index; LV, left ventricle; PAP, pulmonary artery pressure

*Between-group difference from baseline, P value < 0.05 is considered as significant.

### Effect of pioglitazone and empagliflozin on glycemic control and TyG index

Changes in BMI, FBS, HbA1c, Total cholesterol, LDL, HDL, AST, ALT, and TyG index from baseline are shown in Table 4. HbA1c decreased from 8.4 ± 1.1 to 7.0 ± 1.1%, P < 0.01 in the pioglitazone group and from 8.6 ± 1.1 to 7.2 ± 1.0%, P < 0.01 in the empagliflozin group. No statistically significant difference was observed between the two groups by the end of the study, P = 0.78. HDL cholesterol increased significantly in the pioglitazone group but not in the empagliflozin group (P < 0.01). Also, only in the pioglitazone group did the TG level decrease significantly (P < 0.01). Both pioglitazone and empagliflozin significantly reduced the insulin resistance measured by the TyG index at study end, although the difference between the two groups was not significant, (P = 0.16).

**Table 4:** Anthropometric and Biochemical Characteristics of the participants at Baseline and by week 24

	Pioglitazone Group			Empagliflozin Group			
**Variables**	**Baseline** **(n = 36)**	**After 24 weeks** **(n = 36)**	**P value**	**Baseline** **(n = 37)**	**After 24 weeks** **(n = 37)**	**P** ^**^ **value**	P* value between groups
BMI (kg/m^2^)	31.3 ± 5.0	32.1 ± 5.3	0.01	31.0 ± 3.8	29.8 ± 3.6	< 0.01	< 0.01
FBS (mg/dl)	165 ± 49	130 ± 34	< 0.01	177 ± 66	141 ± 46	0.01	0.92
HbA1c (%)	8.4 ± 1.1	7.0 ± 1.1	< 0.01	8.6 ± 1.1	7.2 ± 1.0	< 0.01	0.78
TG (mg/dl)	212.8 ± 127.9	139.4 ± 69.4	< 0.01	161.7 ± 70.6	143.8 ± 83.2	0.21	0.04
Tchol (mg/dl)	158.3 ± 32.4	162.6 ± 34.3	0.56	146.0 ± 30.3	147.6 ± 35.4	0.83	0.79
LDL (mg/dl)	100.1 ± 20.6	83.8 ± 26.9	< 0.01	96.3 ± 26.0	74.5 ± 21.6	< 0.01	0.44
HDL (mg/dl)	46.7 ± 9.3	55.6 ± 9.5	< 0.01	44.8 ± 8.9	46.0 ± 7.5	0.31	< 0.01
ALT (IU/L)	31.5 ± 12.2	21.0 ± 8.6	< 0.01	31.8 ± 17.6	22.2 ± 11.0	< 0.01	0.76
AST (IU/L)	23.0 ± 7.7	19.5 ± 6.5	< 0.01	25.1 ± 14.5	18.8 ± 5.8	< 0.01	0.23
TyG^*^	9.6 ± 0.6	9.0 ± 0.6	< 0.01	9.4 ± 0.7	9.1 ± 0.6	0.01	0.16
**NAFLD fibrosis score**	-1.3 ± 0.9	-0.7 ± 1.0	< 0.01	-1.2 ± 0.8	− 0.9 ± 0.8	0.04	0.04
**FIB4 index**	0.9 ± 0.4	1.0 ± 0.4	0.15	0.9 ± 0.4	0.9 ± 0.3	0.49	0.13

ALT, alanine aminotransferase; AST, aspartate transaminase; BMI, body mass index; CAP, controlled attenuation parameter; FBS, fasting blood sugar; FIB4 index, fibrosis-index; HbA1c, hemoglobin A1c; HDL, high-density lipoprotein; LDL, low-density lipoprotein; LSM, Liver stiffness measurement; NAFLD fibrosis score, non-alcoholoic fatty liver score; Tchol, total Cholesterol; TG, Triglycerides

* Triglyceride-glucose index (TyG) = Ln [FBS (mg/dl) × TG (mg/dl)/2]

TyG index has been proposed as a numerical expression of insulin resistance (IR)

**P value < 0.05 is considered as significant.

*Between-group difference from the baseline, P value < 0.05 is considered as significant.

### Adverse events

Nine non-severe adverse events (AEs) occurred during the study. These included cystitis, gross hematuria, minor hypoglycemia, and lower extremity edema. In addition, two severe AEs happened in two patients, namely, cholangitis and transient ischemic attack that required hospitalization. The severe AEs resolved entirely (Supplement 4).

## Discussion

Left ventricular ejection fraction (LVEF) is a central measure of global systolic function [24]. However, it is influenced by both the intrinsic myocardial function and LV load [25]. GLS is a geometric parameter of LV remodeling, especially at the sub-endocardium fiber level [26]. It measures systolic function and detects subtle myocardial dysfunction.

It is also an independent predictor of CVD, cardiac death, and ventricular arrhythmias [27]. Moreover, impaired GLS is a risk marker of mortality in patients with heart failure with preserved ejection fraction (HFpEF) independent of LVEF and diastolic function [28]. However, we should be cautious about intervening factors on GLS measurements. These include the type of echo equipment, software that provides strain values, and the lack of validated reference values for segmental strain [26].

In patients with diabetes mellitus, abnormal GLS may be a marker of subclinical LV systolic dysfunction, even in asymptomatic patients [29]. On the other hand, CVD is a leading cause of mortality among patients with NAFLD, and current evidence suggests that NAFLD is an independent risk factor for CVD [30]. Recent studies indicate that patients with NAFLD are at an increased risk for incident HFpEF independent of traditional risk factors such as T2DM, body mass index, and hypertension [31, 32].

In the present study, we showed that in patients with T2DM with NAFLD and without established ASCVD, liver fibrosis is associated with reduced GLS at baseline. Our results are in parallel with the CARDIA study [13]. They reported the association of NAFLD with changes in myocardial structure and function and concluded that “NAFLD with T2DM is cross-sectionally associated with subclinical myocardial dysfunction” [7].

We also demonstrated that both empagliflozin and pioglitazone significantly improve liver fibrosis and ameliorate LV remodeling and subtle myocardial dysfunction. However, there was no statistically significant relationship between GLS, LSM, and CAP changes. The relatively small sample size and short duration of the study might act as interfering factors. Moreover, the changes in LSM could be partly related to changes in CAP. Nevertheless, improvement of cardiac structure and function in this study appear to be independent of the improvement of liver steatosis and fibrosis. The greater numerical improvement of GLS in the empagliflozin group compared to pioglitazone has a clinical importance. However, it did not reach the limit of statistical significance. This might be explained by low power of the study to detect statistical significance between the two groups.

Using an animal model, Santos-Gallego et al. showed that empagliflozin in the non-diabetic model reduces LV remodeling and improves myocardial strain by decreasing neuro-hormonal activation. They suggested that the effects of empagliflozin are independent of its impact on glucose homeostasis [33]. In patients with T2DM, delivery of free fatty acid to the liver and its *de-novo* synthesis by the liver is increased [34]. Contribution to worsening insulin resistance, overproduction of fatty acids by visceral adipose tissue, and ectopic accumulation of triglycerides in organs such as the heart and liver. Ng et al. showed that in patients with T2DM, myocardial steatosis is associated with a more significant LV strain [35].

Empagliflozin improves cardiac energetics and metabolism and reduces LV remodeling by increasing the oxidation of free fatty acids, ketone bodies, and branched-chain amino acids [36]. Also, a recent systematic review and meta-analysis showed that SGLT2 inhibition resulted in a significant improvement of LVEF in patients with heart failure, an increase in GLS, and a decrease in LVESV [37]. Their findings confirm the effect of SGLT2 inhibitors on reversing cardiac remodeling. The natriuretic effect of SGLT2 inhibitors is not a driving mechanism of action since an increase in diuresis is transient in patients with T2DM [38]. It seems that a gradual decline of natriuretic peptides might explain the beneficial effects of this class of medications on cardiac function [39]. On the other hand, it has been shown that pioglitazone improves cardiac function in patients with T2DM without ischemic heart disease (IHD) and alters myocardial substrate metabolism [40]. In addition, pioglitazone reduces both myocardial and hepatic triglyceride content [41] and inhibits the inflammatory process that is associated with myocardial fibrosis [40].

The present study demonstrates that pioglitazone improves liver fibrosis and ameliorates subclinical myocardial dysfunction. In addition, empagliflozin and pioglitazone improved diastolic function in our patients with T2DM and NALFD. We showed in our previous studies that empagliflozin effectively improves liver steatosis in patients with and without T2DM [9, 42]. This effect has also been reported in a recent systematic review [43].

In addition, Yoneda et al. evaluated the changes in hepatic steatosis after 24 weeks of pioglitazone and tofogliflozin treatment using MRI-PDFF. No significant differences were observed between the groups concerning relative changes from the baseline [44].

We do have some limitations in our study. It was a single-center study with small number of patients. All patients were asked to follow lifestyle modification recommendations to control HbA1c as an interfering factor. However, although we managed lipid and glycemic control according to the standard guideline, the impact of other unmeasured confounders can never be overemphasized. In addition, we did not perform a liver biopsy as the gold standard method to evaluate liver steatosis and fibrosis.

## Conclusion

Subclinical cardiac dysfunction is highly important in patients with T2DM and with NAFLD. Empagliflozin and Pioglitazone improve LV mechanics and fibrosis in patients without established ASCVD. This has a prognostic importance on cardiovascular outcomes in high-risk patients with T2DM. Moreover, empagliflozin ameliorates liver steatosis more effectively them pioglitazone. This study can serve as a start point hypothesis for the future. Further studies are needed to explore the concept in larger populations.

### Electronic supplementary material

Below is the link to the electronic supplementary material.

Supplementary Material 1

Supplementary Material 2

Supplementary Material 3

Supplementary Material 4

## Data Availability

The datasets used and/or analyzed during the current study available from the corresponding author on reasonable.

## Abbreviations

A4C: Apical 4-chamber view
ADA: American Diabetes Association
ASE: American Society of Echocardiography
ANA: Antinuclear antibodies
ANOVA: Analysis of variance
ASCVD: atherosclerotic cardiovascular disease
BMI: body mass index
CAP: Controlled attenuation parameter
CVD: Cardiovascular disease
DT: Deceleration time
EC: Ethics Committee
EDV: End-diastolic volume
EF: Ejection fraction
eGFR: Estimated glomerular filtration rate
ESV: End-systolic volume
FBS: Fasting blood sugar
GLS: Global longitudinal strain
HbA1c: Glycosylated Hemoglobin, Type A1C
HDL: High-density lipoprotein
HFpEF: Heart failure with preserved ejection fraction
IAGH: Iranian Association of Gastroenterology and Hepatology
IHD: Ischemic heart disease
IPAQ: International Physical Activity Questionnaire
IR: insulin resistance
IRB: Institutional Review Board
IRCT: Iranian Registry of Clinical Trials
ITT: Intention to treat
IVRT: Isovolumic relaxation time
LSM: Liver stiffness measurement
LV: Left ventricular
LVEF: Left ventricular ejection fraction
NAFLD: Non-alcoholic fatty liver disease
NSAIDs: Non-steroidal anti-inflammatory drugs
PAP: Pulmonary artery systolic pressure
PW: Pulsed-wave
SD: Standard deviation
SGLT2: Sodium-glucose Cotransporter-2 (SGLT2) Inhibitors
T2DM: Type 2 diabetes mellitus
TCIR: Time constant of isovolumic relaxation
TDI: Tissue Doppler Imaging
TTE: Transthoracic Echocardiography
TyG index: Triglyceride-glucose index
