# Supplementary material for: Effect of Empagliflozin and Pioglitazone on left ventricular function in patients with type two diabetes and nonalcoholic fatty liver disease without established cardiovascular disease: a randomized single-blind clinical trial
Source: BMC Gastroenterol. 2023 Sep 23;23:327. doi: 10.1186/s12876-023-02948-4 (PMC10517489; doi:10.1186/s12876-023-02948-4)
Supplement: Supplementary file 1 — Supplementary Material 1 [file 12876_2023_2948_MOESM1_ESM.docx]

**Supplement 2.** Patient Enrollment Flowchart


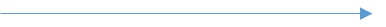

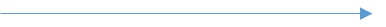

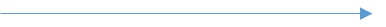

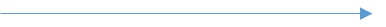

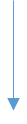

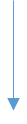

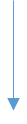


590 T2DM patients entered the screening procedure

30 patients excluded

(CAP<302 dB/m)

2 patients excluded

(EF<50%)

50 patients excluded

(Did not full fill biochemical inclusion and exclusion criteria)

430 patients excluded

(Did not full fill clinical inclusion and exclusion criteria)

Randomization: 73 Patients

Biochemical tests:

160 patients

Elastography: 110 patients

Full filled all inclusion and exclusion criteria: 78 Patients


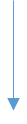

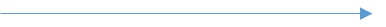

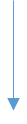

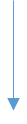

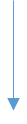


5 patients withdrew their informed consent

Echocardiography: 80 patients

3 patients did not complete the study due to non-adherence to the study medications

Empagliflozin 10mg/day

37 patients

Pioglitazone 30mg/day

36 patients

Completed 24 weeks of treatment

35 patients

Completed 24 weeks of treatment

35 patients
