# Supplementary material for: Effect of Empagliflozin and Pioglitazone on left ventricular function in patients with type two diabetes and nonalcoholic fatty liver disease without established cardiovascular disease: a randomized single-blind clinical trial
Source: BMC Gastroenterol. 2023 Sep 23;23:327. doi: 10.1186/s12876-023-02948-4 (PMC10517489; doi:10.1186/s12876-023-02948-4)
Supplement: Supplementary file 2 — Supplementary Material 2 [file 12876_2023_2948_MOESM2_ESM.docx]

**Supplement 1. Screening procedure**

Screening

7%≤HbA1C≤10.5%

**NO**

**Patient**

**Is excluded.**

**Excluded**

**YES**

ALT or AST >42 in men

ALT or AST>41 in women

OR

ALK > 1.5 *Normal Upper Limit

**YES**

**NO**

**YES**

**Liver Elastography**

CAP score ≥ 302 dB/m

**HBSAg, HCV Ab, ANA, ASMA, TSH, GGT:**

NORMAL

**NO**

**NO**

**YES**

**Patient**

**is excluded.**

**Excluded**

**Patient**

**is excluded.**

**Excluded**

**Echocardiography:**

**EF < 50%**

**OR**

**Wall motion abnormality**

**OR**

**Sign of valvular disease**

**YES**

**NO**

**Patient**

**is enrolled.**

**Excluded**
