# Supplementary material for: Effect of Empagliflozin and Pioglitazone on left ventricular function in patients with type two diabetes and nonalcoholic fatty liver disease without established cardiovascular disease: a randomized single-blind clinical trial
Source: BMC Gastroenterol. 2023 Sep 23;23:327. doi: 10.1186/s12876-023-02948-4 (PMC10517489; doi:10.1186/s12876-023-02948-4)
Supplement: Supplementary file 3 — Supplementary Material 3 [file 12876_2023_2948_MOESM3_ESM.docx]

**Supplement table 3.** Concurrent drug treatment at baseline

| P-value | Empagliflozin | Pioglitazone | Total | Pharmacologic Agent |
| --- | --- | --- | --- | --- |
| 1 | 35 (100%) | 35 (100%) | 70 (100%) | **Statin** (n), (%) |
| 1 | 35 (100%) | 35 (100%) | 70 (100%) | **Metformin** (n), (%) |
| 0.63 | 17 (48.57%) | 19 (54.29%) | 36 (51.43%) | **DPP4 inhibitors** (n), (%) |
| 0.63 | 17 (48.57%) | 19 (54.29%) | 36 (51.43%) | **Sulfonylureas** (n), (%) |
| 1 | 5 (14.29%) | 5 (14.29%) | 10 (14.29%) | **Insulin** (n), (%) |
| 1 | 1 (2.94%) | 1 (2.94%) | 2 (2.94%) | **ACE i** (n), (%) |
| 1 | 12 (35.29%) | 12 (35.29%) | 23 (33.82%) | **ARB** (n), (%) |
| 0.28 | 3 (8.82%) | 6 (17.65%) | 9 (13.24%) | **Beta blocker** (n), (%) |
| 1 | 1 (2.9%) | 1 (2.9%) | 2 (2.9%) | **Diuretics** (n), (%) |
| 0.30 | 3 (8.6%) | 1 (2.9%) | 4 (5%) | **CCB** (n), (%) |

Data are number (%) at baseline
