# Supplementary material for: Effect of Empagliflozin and Pioglitazone on left ventricular function in patients with type two diabetes and nonalcoholic fatty liver disease without established cardiovascular disease: a randomized single-blind clinical trial
Source: BMC Gastroenterol. 2023 Sep 23;23:327. doi: 10.1186/s12876-023-02948-4 (PMC10517489; doi:10.1186/s12876-023-02948-4)
Supplement: Supplementary file 4 — Supplementary Material 4 [file 12876_2023_2948_MOESM4_ESM.docx]

**Supplement 4.** Adverse Events during the study

| Empagliflozin | Pioglitazone | Adverse events |
| --- | --- | --- |
| 1 | 0 | Transient Ischemic Attack |
| 0 | 2 | Minor Hypoglycemia |
| 1 | 0 | Cholangitis |
| 0 | 5 | Lower extremity edema |
| 1 | 0 | Cystitis |
| 1 | 0 | Gross hematuria |
